# Associations of Prenatal and Childhood Antibiotic Exposure With Obesity at Age 4 Years

**DOI:** 10.1001/jamanetworkopen.2019.19681

**Published:** 2020-01-22

**Authors:** Karen S. W. Leong, Jessica McLay, José G. B. Derraik, Sheree Gibb, Nichola Shackleton, Rachael W. Taylor, Marewa Glover, Rick Audas, Barry Taylor, Barry J. Milne, Wayne S. Cutfield

**Affiliations:** 1 Liggins Institute, University of Auckland, Auckland, New Zealand; 2 A Better Start National Science Challenge, Auckland, New Zealand; 3 Centre of Methods and Policy Application in the Social Sciences, University of Auckland, Auckland, New Zealand; 4 Department of Women’s and Children’s Health, Uppsala University, Uppsala, Sweden; 5 Dunedin School of Medicine, University of Otago, Dunedin, New Zealand; 6 Centre of Research Excellence, Indigenous Sovereignty and Smoking, Auckland, New Zealand

## Abstract

**Importance:**

Although antibiotics are associated with obesity in animal models, the evidence in humans is conflicting.

**Objective:**

To assess whether antibiotic exposure during pregnancy and/or early childhood is associated with the development of childhood obesity, focusing particularly on siblings and twins.

**Design, Setting, and Participants:**

This cross-sectional national study included 284 211 participants (132 852 mothers and 151 359 children) in New Zealand. Data analyses were performed for 150 699 children for whom data were available, 30 696 siblings, and 4188 twins using covariate-adjusted analyses, and for 6249 siblings and 522 twins with discordant outcomes using fixed-effects analyses. Data analysis was performed November 2017 to March 2019.

**Exposure:**

Exposure to antibiotics during pregnancy and/or early childhood.

**Main Outcomes and Measures:**

The main outcome is odds of obesity at age 4 years. Anthropometric data from children born between July 2008 and June 2011 were obtained from the B4 School Check, a national health screening program that records the height and weight of 4-year-old children in New Zealand. These data were linked to antibiotics (pharmaceutical records) dispensed to women before conception and during all 3 trimesters of pregnancy and to their children from birth until age 2 years.

**Results:**

The overall study population consisted of 132 852 mothers and 151 359 children (77 610 [51.3%] boys) who were aged 4 to 5 years when their anthropometrical measurements were assessed. Antibiotic exposure was common, with at least 1 course dispensed to 35.7% of mothers during pregnancy and 82.3% of children during the first 2 years of life. Results from covariate-adjusted analyses showed that both prenatal and early childhood exposures to antibiotics were independently associated with obesity at age 4 years, in a dose-dependent manner. Every additional course of antibiotics dispensed to the mothers yielded an adjusted odds ratio (aOR) of obesity in their children (siblings) of 1.02 (95% CI, 0.99-1.06), which was similar to the odds across pregnancy for the whole population (aOR, 1.06; 95% CI, 1.04-1.07). For the child’s exposure, the aOR for the association between antibiotic exposure and obesity was 1.04 (95% CI, 1.03-1.05) among siblings and 1.05 (95% CI, 1.02-1.09) among twins. However, fixed-effects analyses of siblings and twins showed no associations between antibiotic exposure and obesity, with aORs of 0.95 (95% CI, 0.90-1.00) for maternal exposure, 1.02 (95% CI, 0.99-1.04) for child’s exposure, and 0.91 (95% CI, 0.81-1.02) for twins’ exposure.

**Conclusions and Relevance:**

Although covariate-adjusted analyses demonstrated an association between antibiotic exposure and odds of obesity, further analyses of siblings and twins with discordant outcomes showed no associations. Thus, these discordant results likely reflect unmeasured confounding factors.

## Key Points

**Question:**

Is antibiotic exposure during pregnancy and/or during early childhood associated with the development of childhood obesity?

**Findings:**

This cross-sectional national study of 284 211 participants (132 852 mothers and 151 359 children) in New Zealand found that both prenatal and early childhood exposures to antibiotics were independently associated with obesity at age 4 years in a dose-dependent manner. However, fixed-effects analyses of siblings and twins with discordant outcomes showed no associations between antibiotic exposure and obesity.

**Meaning:**

Although judicious use of antibiotics is necessary, antibiotics are unlikely to be a major contributor to childhood obesity.

## Teaser

This cross-sectional study of mothers and their children in New Zealand assesses whether exposure to antibiotics during pregnancy and/or early childhood is associated with the development of childhood obesity.

## Introduction

Historically, antibiotics have been used as growth-promoting agents in farm animals,^1^ and research in animals has shown that antibiotics are associated with obesity in mice.^2,3^ However, evidence in humans is still inconsistent. In children, several studies^4,5,6,7^ have reported that antibiotics are not associated with the development of childhood obesity. Conversely, numerous studies,^8,9,10,11^ including cross-sectional and cohort studies, have reported a positive association between antibiotic exposure and development of childhood obesity. Most of those studies looked at the association between postnatal exposure and obesity, and a limited number examined the association between prenatal exposure and obesity.^11^

However, there is concern that previous positive studies have not adequately accounted for potential confounders, including genetic and environmental factors.^8,9,10^ In particular, very few studies have examined siblings or twins to account for possible unmeasured confounders, and the studies that did include these analyses reported no associations between antibiotic exposure and risk of childhood obesity.^5,6,12^ Nonetheless, these studies were not without limitations, because they consisted of small cohorts and did not include national data or analyses on both siblings from singleton pregnancies (henceforth referred to as “siblings”) and those from multiple pregnancies (henceforth referred to as “twins”).

Therefore, we used national data from New Zealand to assess whether antibiotic exposure among women during pregnancy and/or among their children early in life was associated with the likelihood of childhood obesity at age 4 years. Furthermore, to address the confounding influence of environmental and genetic factors, we examined these associations among siblings and twins.

## Methods

This study was approved by the University of Otago Human Ethics Committee. Informed consent was not obtained as per Rule 11(2)(c)(iii) of the Health Information Privacy code,^13^ which allows for anonymized health data to be used for research purposes without the authorization of the individual concerned, under certain circumstances. The New Zealand Ministry of Health has confirmed that these circumstances are met for use of the B4 School Check data within the New Zealand’s Integrated Data Infrastructure.^14^ This study follows the Strengthening the Reporting of Observational Studies in Epidemiology (STROBE) reporting guideline.^15^

### Study Design and Participants

We conducted a retrospective cross-sectional observational study consisting of 151 359 children and 132 852 mothers. These children were born between July 2008 and June 2011, and they participated in the B4 School Check between July 2012 and June 2016 when they were 4 to 5 years old. The B4 School Check is a national screening program that was established in September 2008 to improve health and promote well-being in preschool children in New Zealand.^14^

### Exposure

By use of the New Zealand Integrated Data Infrastructure, which is a collection of whole-population administrative data sources linked at the individual level,^16^ we linked children’s birth records to their and their mothers’ pharmaceutical records. Antibiotic exposure was measured via pharmaceutical dispensing records managed by the New Zealand Pharmaceutical Management Agency, which are available from July 1, 2007.^17^ We obtained information on the number of courses of antibiotics dispensed among pregnant women per trimester and during the 3 months before conception. Antibiotic exposure among their children was recorded from birth until age 2 years and was examined as total exposure and as exposure within 3 periods: birth to 6 months, 6 to 12 months, and 12 to 24 months. Exposure was also categorized by narrow-spectrum (effective against gram-positive bacteria) and broad-spectrum (effective against both gram-positive and gram-negative bacteria) antibiotics. To negate the effect of a long tail in our data set, we truncated the distribution of number of antibiotic courses as follows: for mothers, 5 or more for overall pregnancy, 3 or more for each trimester, and 3 or more the preconception period; for children, 10 or more for the first 2 years of life, 5 or more from birth to 6 months, 5 or more from 6 to 12 months, and 10 or more from 12 to 24 months; for broad-spectrum antibiotics, 5 or more across the whole pregnancy and 10 or more for the child’s first 2 years of life; and for narrow-spectrum antibiotics, 2 or more across the whole pregnancy and 4 or more for the child’s first 2 years of life. Children and their mothers who were administered intravenous antibiotics were excluded (<100 cases) because receiving intravenous antibiotics would likely indicate a more serious medical condition that could have a marked association with weight.

### Outcomes

The child’s height and weight were measured by registered nurses or nurse practitioners. Height was measured to the nearest 0.1 cm with a portable stadiometer (either Leicester Height Measure or a SECA 214), and weight was measured to the nearest 100 g using a floor scale (SECA 862, Tanita WB 100 S MA, SECA 770, or Tanita HD-351) that was calibrated at least twice per year. Body mass index (BMI) was calculated as the weight in kilograms divided by height in meters squared and converted into sex- and age-specific BMI *z* scores using the World Health Organization’s Anthro macro (version 3.2.2) for Stata statistical software. Children with a BMI *z* score at or above the 95th percentile were classified as children with obesity.

### Confounders

The child’s year and month of birth, birth weight, gestational age, delivery mode, birth order, and an indicator for multiple births (eg, twins and triplets) were obtained from the birth records, as was maternal age. We created a binary indicator of low birth weight (<2500 g) and normal-to-high birth weight (≥2500 g) and categorized length of gestation into very preterm (<32 weeks), preterm (≥32 to <37 weeks), term (≥37 to <42 weeks), and postterm (≥42 weeks). Maternal age was categorized into 6 groups (<20, 20-24, 25-29, 30-34, 35-39, and ≥40 years). Delivery mode was categorized as unassisted vaginal birth, assisted vaginal birth, cesarean delivery, and unknown. Birth order was dichotomized as first or later birth.

Indicators for gestational diabetes and hyperemesis gravidarum were derived from data on publicly funded hospital records. From the same records, an indicator of maternal or child health was constructed as a binary variable according to whether the mother (during pregnancy) or child (during the first 2 years of life) spent 3 or more consecutive days in the hospital. Information on socioeconomic status and urban or rural location was based on residential address at the time of the B4 School check. Socioeconomic status was estimated using the New Zealand Index of Deprivation 2013,^18^ which was derived from the socioeconomic deprivation characteristics of “meshblocks,” which are small areas with a typical population of 60 to 110 people. This assigned each individual to 1 of 10 deciles, ranging from 1 (least socioeconomically deprived) to 10 (most socioeconomically deprived). Ethnicity was determined from the source ranked ethnicity table in the New Zealand Integrated Data Infrastructure,^16^ which prioritizes reports from the census (the best-quality ethnicity information), followed by birth records, and then administrative sources. Individuals were classified into 1 or more of the following major ethnic groups: European; Māori; Pacific; Asian; Middle Eastern, Latin American, or African; and other. In compliance with the statistical standard for reporting ethnicity in New Zealand, ethnic groups were not mutually exclusive.^19^

### Statistical Analysis

#### Statistical Analyses With Covariate Adjustment

Unadjusted associations between the number of courses of antibiotics and obesity at age 4 years were assessed for maternal antibiotic exposure throughout pregnancy and during each trimester of pregnancy, as well as during the 3 months before conception, and for child exposure during the first 2 years of life and for the periods birth to 6 months, 6 to 12 months, and 12 to 24 months.

We used multiple logistic regression to assess the adjusted association between the number of courses of antibiotics and childhood obesity at age 4 years, controlling for a number of confounders. Similarly, multiple linear regressions controlling for a number of confounders were used to examine associations with BMI *z* score at age 4 years. Separate analyses were conducted for the same periods investigated in the unadjusted analyses (as itemized already). We ran separate analyses on the number of courses of either narrow-spectrum or broad-spectrum antibiotics across pregnancy for the mothers and in the first 2 years of life for the children. Standard errors are adjusted for clustering by mother using a sandwich estimator.^20^

#### Fixed-Effects Analyses: Siblings

Siblings were identified in the data set by maternal identification. Only siblings born between July 2008 and June 2011 were included. We used a fixed-effects model with siblings nested within mothers, in which only the variance within mothers (between siblings) is used to assess the association of exposure to antibiotics with child obesity. These models assess potential differences in obesity prevalence between siblings, where 1 sibling was exposed to more antibiotic courses prenatally, and also where 1 sibling was exposed to more antibiotic courses during infancy. These models control for unmeasured confounders consistent between siblings, such as maternal characteristics and aspects of the home environment. Nonetheless, models controlled for the same confounders as before, with the exception of ethnicity and socioeconomic status, which vary little between siblings. Because the fixed-effect analyses exploit within-mother variance, only siblings with different values for the outcome are included in the analysis. Thus, the sample size when considering obesity (a binary variable) was smaller than in analyses examining BMI *z* scores.

#### Fixed-Effects Analyses: Twins

Twins were identified according to date of birth and maternal identification. We used fixed-effects models with twins nested within mothers, which allowed us to control for a greater amount of unmeasured confounding, including factors within a given pregnancy. Therefore, we controlled only for measured characteristics that could differ between twins: sex, birth weight, length of hospital stay (≥3 consecutive days vs no), and total number of days overseas. Like the sibling analysis, only twins with different values for the outcome were included for childhood obesity.

#### Additional Covariate Analyses

We undertook the same covariate-adjusted analyses on the cohort of siblings and twins (ie, without fixed effects controlling for unmeasured confounding) to test whether associations were similar to those for the whole population. Observing similar results would provide some confidence that any differences between the covariate-adjusted analyses on the whole population and the fixed-effects analyses on the siblings and twins were due to the additional confounders rather than any differences between the whole population and siblings or twins cohort. *P* values were not calculated. All analyses were undertaken using Stata statistical software version 15.1 (StataCorp). Data analysis was performed November 2017 to March 2019.

## Results

Our study population consisted of 284 211 participants: 132 852 mothers and 151 359 children (77 610 [51.3%] boys). Data from 150 699 children were used in the analyses. Nearly all of the children were singleton births (146 850 [97.0%]), 77 769 (51.4%) were first births, 142 563 (94.2%) were of normal-to-high birth weight, 139 347 (92.1%) were full term, and 36 291 children (24.0%) were delivered by cesarean delivery (Table 1). The children were aged 4 to 5 years when their anthropometrical measurements were assessed. There were 23 922 children (15.8%) with obesity, with a mean (SD) BMI *z* score of 0.68 (1.05). Further description of the characteristics of our study population are shown in Table 1 and eTable 1 in the Supplement.

**Table 1.** Demographic Characteristics of the Whole Population^a^

Characteristic	Participants, No. (%)
Mothers (n = 132 852)	
Age at childbirth, y	
<20	10 296 (6.8)
20-24	26 928 (17.8)
25-29	37 377 (24.7)
30-34	42 834 (28.3)
35-39	27 693 (18.3)
≥40	6225 (4.1)
Parity	
First birth	77 769 (51.4)
Later birth	73 590 (48.6)
Socioeconomic status, by New Zealand Index of Deprivation 2013 quintile	
1 (Least socioeconomically deprived)	28 266 (18.7)
2	27 645 (18.3)
3	27 840 (18.4)
4	29 463 (19.5)
5 (Most socioeconomically deprived)	37 890 (25.0)
Gestational diabetes	5061 (3.3)
Hyperemesis gravidarum	2826 (1.9)
≥3 d in hospital during pregnancy	17 811 (11.8)
Children (n = 151 359)	
Year of birth	
2008	24 114 (15.9)
2009	49 803 (32.9)
2010	51 933 (34.3)
2011	25 509 (16.9)
Sex	
Male	77 610 (51.3)
Female	73 749 (48.7)
Ethnicity	
European	106 866 (70.6)
Māori	41 481 (27.4)
Pacific	21 570 (14.3)
Asian	18 444 (12.2)
Middle Eastern, Latin American, or African	2223 (1.5)
Other	2301 (1.5)
Birth weight	
Low (<2500 g)	8532 (5.6)
Normal to high (≥2500 g)	142 563 (94.2)
Gestational age	
Very preterm (<32 wk)	1554 (1.0)
Preterm (≥32 to <37 wk)	9435 (6.2)
Term (≥37 to <42 wk)	139 347 (92.1)
Postterm (≥42 wk)	1026 (0.7)
Delivery mode	
Unassisted vaginal	90 195 (59.6)
Assisted vaginal	13 512 (8.9)
Cesarean	36 291 (24.0)
Unknown	11 358 (7.5)
Birth	
Singletons	146 850 (97.0)
Twins or triplets	4509 (3.0)
≥3 d in hospital in first 2 y of life	17 811 (11.8)
Children with obesity	23 922 (15.8)

^a^ The denominator for all percentages provided in the table is the number of children or mothers in the cohort.

### Antibiotic Exposure

Table 2 shows the number of courses of antibiotics dispensed to mothers during the preconception period and throughout pregnancy, and to children during the first 2 years of life. In total, 35.7% of mothers were dispensed at least 1 course of antibiotics during pregnancy, with 1.2% dispensed 5 or more courses. Rates of antibiotics dispensed to mothers were similar during preconception period and throughout pregnancy (Table 2).

**Table 2.** Courses of Antibiotics Dispensed to Mothers and Their Children

Antibiotic Courses, No.	Participants, No. (%)								
	Mothers					Children			
	Preconception^a^^,^^b^	Trimester^b^			Across Pregnancy^b^	Age, mo			
		First	Second	Third		0-5.9^b^	6-11.9^b^	12-24	0-24
0	128 310 (84.8)	128 031 (84.6)	127 953 (84.5)	126 741 (83.7)	97 278 (64.3)	117 756 (77.8)	76 707 (50.7)	41 130 (27.2)	26 853 (17.7)
1	17 433 (11.5)	18 138 (12.0)	18 282 (12.1)	19 104 (12.6)	32 421 (21.4)	21 963 (14.5)	35 082 (23.2)	32 553 (21.5)	25 041 (16.5)
2	4134 (2.7)	3966 (2.6)	3882 (2.6)	4170 (2.8)	12 621 (8.3)	7050 (4.7)	18 468 (12.2)	24 420 (16.1)	21 486 (14.2)
3	1479 (1.0)	1224 (0.8)	1242 (0.8)	1347 (0.9)	5052 (3.3)	2583 (1.7)	9843 (6.5)	16 989 (11.2)	17 391 (11.5)
4	NA	NA	NA	NA	2196 (1.5)	1014 (0.7)	5268 (3.5)	11 583 (7.7)	13 833 (9.1)
5	NA	NA	NA	NA	1788 (1.2)	993 (0.7)	5994 (4.0)	7977 (5.3)	10 722 (7.1)
6	NA	NA	NA	NA	NA	NA	NA	5388 (3.6)	8370 (5.5)
7	NA	NA	NA	NA	NA	NA	NA	3723 (2.5)	6273 (4.1)
8	NA	NA	NA	NA	NA	NA	NA	2502 (1.7)	4896 (3.2)
9	NA	NA	NA	NA	NA	NA	NA	1665 (1.1)	3795 (2.5)
≥10	NA	NA	NA	NA	NA	NA	NA	3435 (2.3)	12 696 (8.4)

Abbreviation: NA, not applicable.

^a^ The preconception period refers to the 3 months before pregnancy.

^b^ Number of courses was truncated at 5 or more for all periods except for the child’s exposure at 12 to 24 months and in the first 2 years of life.

Among the children, 82.3% were dispensed at least 1 course of antibiotics within the first 2 years of life. Notably, 30.8% of children were dispensed at least 5 courses, with 8.4% dispensed 10 or more courses. Antibiotics were most commonly dispensed in the second year of life, when nearly three-quarters of all children (72.8%) were dispensed at least 1 course, followed by the period from 6 to 12 months (49.3%), and then the first 6 months of life (22.2%) (Table 2). For both mothers and children, broad-spectrum antibiotics were approximately 4 times more likely to be dispensed than narrow-spectrum antibiotics (eTable 2 in the Supplement).

### Antibiotics and Obesity

For both mothers during pregnancy and children during the first 24 months of life, more antibiotic courses were associated with increasing prevalence of obesity at age 4 years (Figure). For mothers, this trend was observed at each trimester during pregnancy: the prevalence of obesity increased from 17.1% with 1 antibiotic prescription to 22.3% with 5 antibiotic prescriptions during pregnancy (Figure). In contrast, the prevalence of obesity remained steady with increasing prescriptions during the preconception period (eFigure 1 in the Supplement). With regard to childhood antibiotic exposure, there were clear dose-response associations between number of antibiotic courses in each of the periods examined (birth to 6 months, 6 to 12 months, and 12 to 24 months) and obesity prevalence at age 4 years (Figure). In addition, increasing numbers of either broad-spectrum or narrow-spectrum antibiotics dispensed for both mothers and children were associated with greater prevalence of obesity (eFigure 2 in the Supplement).

**Figure.**
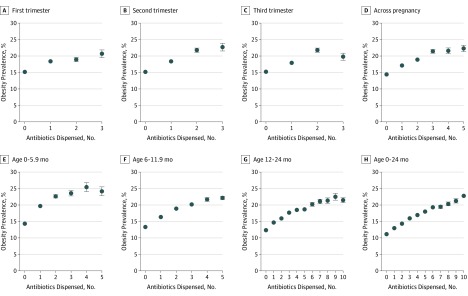
Prevalence of Obesity by Number of Antibiotic Courses Dispensed Graphs show prevalence of obesity according to number of antibiotic courses dispensed by pregnancy trimester (first [A], second [B], third [C], and entire pregnancy [D]) and age of child (birth to 5.9 months [E], 6-11.9 months [F], 12-24 months [G], and birth to 24 months [H]). Error bars denote standard errors.

These descriptive findings as shown in the Figure were confirmed in covariate-adjusted analyses, with clear independent associations between greater maternal and child’s exposure to antibiotics and increased obesity prevalence and BMI *z* score (Table 3). Associations were apparent in every trimester of pregnancy (first trimester, adjusted odds ratio [aOR] = 1.05 [95% CI, 1.02-1.08] and β = 0.024 [95% CI, 0.014-0.035]; second trimester, aOR = 1.11 [95% CI, 1.08-1.14] and β = 0.039 [95% CI, 0.029-0.050]; third trimester, aOR = 1.05 [95% CI, 1.03-1.08] and β = 0.034 [95% CI, 0.024-0.044]), but not during preconception (aOR = 1.02 [95% CI, 0.99-1.05] and β = 0.010 [95% CI, 0.000-0.020]). Associations were also apparent during the 3 periods examined in the first 2 years of life (birth to 6 months, aOR = 1.08 [95% CI, 1.06-1.10] and β = 0.037 [95% CI, 0.031-0.044]; 6 to 12 months, aOR = 1.07 [95% CI, 1.06-1.08] and β = 0.035 [95% CI, 0.031-0.039]; 12 to 24 months, aOR = 1.06 [95% CI, 1.05-1.07] and β = 0.028 [95% CI, 0.025-0.031]). Associations were also apparent for both maternal and child’s exposure to either broad-spectrum or narrow-spectrum antibiotics (eTable 3 and eTable 4 in the Supplement).

**Table 3.** Associations of Maternal and Child’s Antibiotic Exposure With Obesity and BMI *z *Score at Age 4 Years^a^

Timing of Antibiotic Use	Obesity, aOR (95% CI)	BMI *z* Score, Adjusted β Coefficient (95% CI)
Maternal exposure		
3 mo before conception	1.02 (0.99-1.05)	0.010 (0.000-0.020)
First trimester	1.05 (1.02-1.08)	0.024 (0.014-0.035)
Second trimester	1.11 (1.08-1.14)	0.039 (0.029-0.050)
Third trimester	1.05 (1.03-1.08)	0.034 (0.024-0.044)
Any time in pregnancy	1.06 (1.04-1.07)	0.026 (0.020-0.031)
Child’s exposure		
Birth to 5.9 mo	1.08 (1.06-1.10)	0.037 (0.031-0.044)
6-11.9 mo	1.07 (1.06-1.08)	0.035 (0.031-0.039)
12-24 mo	1.06 (1.05-1.07)	0.028 (0.025-0.031)
Birth to 24 mo	1.04 (1.04-1.05)	0.019 (0.018-0.021)

Abbreviations: aOR, adjusted odds ratio; BMI, body mass index (calculated as the weight in kilograms divided by height in meters squared).

^a^ Reported estimates represent the association of 1 additional course of antibiotics. Models were adjusted for birth year and month, child sex and ethnicity, maternal age, parity, birth weight, gestational age, delivery mode, multiple birth status, maternal diabetes and hyperemesis gravidarum, prolonged (≥3 days) stay in hospital during pregnancy and during the first 24 months of the child’s life, neighborhood socioeconomic deprivation, rural vs urban states, number of days overseas during pregnancy (mother analyses only), and number of days overseas during the first 24 months of life (child analyses only).

### Siblings and Twins

Results from analyses conducted on 30 696 siblings and 4188 twins are shown in Table 4. In covariate-adjusted analyses (which mimicked analyses conducted on the whole population), every additional course of antibiotics dispensed to the mothers yielded an aOR of obesity in their children (siblings) of 1.02 (95% CI, 0.99-1.06) (Table 4), which was similar to the odds across pregnancy for the whole population (aOR, 1.06; 95% CI, 1.04-1.07) (Table 3). For the child’s exposure, the aOR for the association between antibiotic exposure and obesity was 1.04 (95% CI, 1.03-1.05) among siblings and 1.05 (95% CI, 1.02-1.09) among twins (Table 4), similar to the overall population (aOR, 1.04; 95% CI, 1.04-1.05) (Table 3).

**Table 4.** Associations of Maternal and Child’s Antibiotic Exposure With Obesity and BMI *z* Score at Age 4 Years by Siblings and Twins^a^

Antibiotic Exposure	Obesity, aOR (95% CI)		BMI *z* Score, Adjusted β Coefficient (95% CI)	
	Covariate Adjusted	Family Fixed Effects^b^	Covariate Adjusted	Family Fixed Effects
Siblings, No.	30 696	6249	30 696	30 696
Maternal exposure	1.02 (0.99 to 1.06)	0.95 (0.90 to 1.00)	0.017 (0.006 to 0.028)	−0.008 (−0.024 to 0.008)
Child’s exposure	1.04 (1.03 to 1.05)	1.02 (0.99 to 1.04)	0.017 (0.013 to 0.020)	0.006 (0.000 to 0.012)
Twins, No.	4188	522	4188	4188
Child’s exposure	1.05 (1.02 to 1.09)	0.91 (0.81 to 1.02)	0.018 (0.008 to 0.028)	−0.011 (−0.026 to 0.005)

Abbreviations: aOR, adjusted odds ratio; BMI, body mass index (calculated as the weight in kilograms divided by height in meters squared).

^a^ Reported estimates represent the association of 1 additional course of antibiotics any time in pregnancy for the mother and any time in the first 24 months of life for the child. Sibling analyses were adjusted for sex, birth weight, maternal diabetes and hyperemesis gravidarum, birth order, gestational age, prolonged (≥3 days) stay in hospital during pregnancy and during the first 24 months of the child’s life, birth year and month, and total number of days overseas. Twin analyses were adjusted for sex, birth weight, prolonged (≥3 days) stay in hospital during the first 24 months of the child’s life, and total number of days overseas.

^b^ Where obesity was the outcome, only siblings and twins with discordant outcomes (ie, 1 with obesity and 1 without it) were included in the analyses accounting for family fixed effects.

However, these associations disappeared in fixed-effects analyses of 6249 siblings and 522 twins with discordant outcomes, which accounted for unmeasured family-level confounders. Thus, there were no statistically significant associations between antibiotic exposure and the odds of obesity for both maternal (aOR, 0.95; 95% CI, 0.90 to 1.00) and child’s (aOR, 1.02; 95% CI, 0.99 to 1.04) exposure in the sibling cohort, and child’s exposure in the twin cohort (aOR, 0.91; 95% CI, 0.81 to 1.02) (Table 4). Similarly, there were no statistically significant associations between antibiotics and BMI *z* score for either maternal (β, −0.008; 95% CI −0.024 to 0.008) or child’s (β, 0.006; 95% CI, 0.000 to 0.012) exposure in the sibling cohort, or child’s exposure in the twin cohort (β, −0.011; 95% CI, −0.026 to 0.005) (Table 4).

## Discussion

The results of this study indicate that both the mother’s and child’s exposure to antibiotics were independently associated with obesity among children at age 4 years, in a dose-dependent manner. Although the aORs for our study were small, these results were comparable to findings from previous studies. Two systematic reviews and meta-analyses^8,9^ of studies investigating the association between antibiotic exposure in early childhood and odds of childhood obesity revealed pooled odds ratios of 1.05 (95% CI, 1.00-1.11) and 1.11 (95% CI, 1.02-1.20), respectively. This is the reason for further analyses in our study to look at possible confounders that may influence these results. Our analyses of siblings and twins (fixed-effects analyses) showed that there were no statistically significant associations between prenatal and early childhood antibiotic exposure and the odds of developing childhood obesity.

An important point to note is that the covariate-controlled analyses of sibling and twins showed largely the same magnitude of associations between antibiotic exposure and childhood obesity as with the whole population. This suggests that the fixed-effects analyses produced null findings because of their ability to control for unmeasured confounding. This limitation was raised in previous studies^6^ showing positive associations, whereby it was difficult to pinpoint antibiotic use as the primary factor associated with obesity without considering other confounding factors, such as host genetics, maternal BMI, environmental factors, and infection itself. The findings of the current study suggest that unmeasured confounding may have played a role in the associations found in previous studies.

In the fixed-effects analyses undertaken in the current study, most factors shared within families would have been controlled for, including, for example, the home environment for siblings and both the home and in utero environments for twins. Our findings are consistent with those of previous studies.^5,6,12^ For example, in a small study of 46 twin pairs, Gerber et al^5^ reported that antibiotic exposure within the first 6 months of life was not associated with weight gain during early childhood. In a larger study of 547 twin pairs, Li et al^6^ reported no association between antibiotic exposure and risk of childhood obesity. Together, our findings raise questions regarding what unmeasured factors might explain the apparent associations between antibiotic exposure and obesity in our covariate-adjusted analyses.

Obesity is a condition resulting from a complex interaction of genetic, diet, and lifestyle factors. Numerous factors have been found to be associated with the risk of childhood obesity, as reported in a systematic review,^21^ including higher maternal prepregnancy BMI, excess gestational weight gain, and accelerated infant weight gain. When controlling for maternal BMI, 1 study^7^ showed no association between maternal antibiotics use and childhood obesity. Unfortunately, this information is not readily available in the New Zealand Integrated Data Infrastructure and could not be included in our analyses. In addition, both maternal and infant weight gain are associated with dietary habits and practices among mothers.^22^ Another significant risk factor for childhood obesity is lower socioeconomic status, which is associated with higher rates of antibiotic prescriptions, higher prevalence of smoking, and childhood obesity.^23,24^

Associations between antibiotic use and obesity are postulated to be due to perturbations of the gut microbiome.^25^Antibiotics lead to dysbiosis, which, in turn, has been proposed to lead to development of obesity.^25^ The mechanisms by which antibiotics indirectly modulate weight gain are unclear, but there are a number of hypotheses,^26,27^ including an increased ability of gut bacteria to extract energy from indigestible polysaccharides, a reduction in the number of bacteria that are metabolically protective against obesity, altered hepatic lipogenesis, altered metabolic signaling, and a reduction in intestinal defense and immunity.

One speculative explanation for our discordant findings on the whole population and the siblings and twins analyses beyond confounding may be sharing of the untreated sibling’s microbiome (ie, fecal-oral transfer). In this scenario, the dysbiosis in the sibling treated with antibiotics could have been corrected by cross-transfer of microbiome from the untreated sibling. Evidence from animal studies supports this concept. Ridaura et al^28^ showed that cohousing of both recipient mice harboring either the lean or obesity microbiota from human donors prevented an increase in adiposity in the mice who received the obesity microbiota. It appeared that the recipient of the lean microbiota played a role in protecting against obesity in those who received the obesity microbiota.^28^

### Limitations

The major limitation of this study (as with all other preceding studies on this topic) is its retrospective nonrandomized study design, which is unable to determine the causative relationship between early antibiotic exposure and childhood obesity. However, prospective randomized studies on the association between early antibiotic exposure and childhood obesity cannot be performed because they would be unethical. Other limitations to the study include a lack of information on parental BMI (in particular maternal BMI), which is associated with the development of childhood obesity.^29^ Information on short courses of antibiotics, typically single-dose antibiotic administration during the peripartum period, was not available for our analyses, and information on the number of antibiotics dispensed was considered equivalent to antibiotic exposure. Furthermore, we were unable to obtain accurate information on indications for antibiotic prescriptions, actual use, and compliance.

Another limitation of this study is the sample size for the obesity outcome among the siblings and twins. The fixed-effects analyses of obesity outcomes for siblings and twins, which demonstrated a null finding, were restricted to siblings and twins who differed on obesity status and so involved a far smaller sample. However, we note that similar null findings were seen in our fixed-effects analyses of BMI *z* score for siblings and twins, for which the sample size remained the same. Nonetheless, a strength of our study was the use of national data and our large sample size of 151 359 children and their mothers, which is one of the largest studies ever conducted on the associations between antibiotic exposure and childhood obesity. Importantly, with this large cohort, we were able to conduct further subgroup analyses on siblings and twins to better account for confounding factors.

## Conclusions

Although initial analyses on the whole population, siblings, and twins demonstrated an association between antibiotic exposure and odds of obesity, further detailed analyses of siblings and twins with discordant outcomes showed no associations. These discordant results likely reflect unmeasured confounding factors. Therefore, although judicious use of antibiotics is necessary, antibiotics are unlikely to be a major contributor to childhood obesity.

## Notes

Supplement. **eFigure 1. **Obesity Prevalence at Age 4 Years According to Number of Antibiotics Dispensed to Mother in the Pre-conception Period (3 Months Before Pregnancy) **eFigure 2. **Obesity Prevalence at Age 4 Years According to Number of Broad or Narrow Spectrum Antibiotics Dispensed **eTable 1. **Demographic Characteristics of the Siblings (n = 35 994) and Twins (n = 4362) Cohort **eTable 2. **Courses of Broad and Narrow Spectrum Antibiotics Dispensed to Mothers During Pregnancy, and to Children During the First 24 Months of Life **eTable 3. **Associations Between Maternal and Child’s Exposure to Narrow and Broad Spectrum Antibiotics and Obesity and BMI *z*-Score at Age 4 Years **eTable 4. **Classification of Antibiotics According to Spectrum of Activity
