# Supplementary material for: Associations of Prenatal and Childhood Antibiotic Exposure With Obesity at Age 4 Years
Source: JAMA Netw Open. 2020 Jan 22;3(1):e1919681. doi: 10.1001/jamanetworkopen.2019.19681 (PMC6991276; doi:10.1001/jamanetworkopen.2019.19681)
Supplement: Supplement. — eFigure 1. Obesity Prevalence at Age 4 Years According to Number of Antibiotics Dispensed to Mother in the Pre-conception Period (3 Months Before Pregnancy) eFigure 2. Obesity Prevalence at Age 4 Years According to Number of Broad or Narrow Spectrum Antibiotics Dispensed eTable 1. Demographic Characteristics of the Siblings (n = 35 994) and Twins (n = 4362) Cohort eTable 2. Courses of Broad and Narrow Spectrum Antibiotics Dispensed to Mothers During Pregnancy, and to Children During the First 24 Months of Life eTable 3. Associations Between Maternal and Child’s Exposure to Narrow and Broad Spectrum Antibiotics and Obesity and BMI z-Score at Age 4 Years eTable 4. Classification of Antibiotics According to Spectrum of Activity [file jamanetwopen-3-e1919681-s001.pdf]

Supplementary Online Content

Leong KSW, McLay J, Derraik JGB, et al. Associations of prenatal and childhood antibiotic exposure with obesity at age 4 years. *JAMA Netw Open*. 2020;3(1):e1919681. doi:10.1001/jamanetworkopen.2019.16981

eFigure 1. Obesity Prevalence at Age 4 Years According to Number of Antibiotics Dispensed to Mother in the Pre-conception Period (3 Months Before Pregnancy)

eFigure 2. Obesity Prevalence at Age 4 Years According to Number of Broad or Narrow Spectrum Antibiotics Dispensed

eTable 1. Demographic Characteristics of the Siblings (n=35 994) and Twins (n=4362) Cohort

eTable 2. Courses of Broad and Narrow Spectrum Antibiotics Dispensed to Mothers During Pregnancy, and to Children During the First 24 Months of Life

eTable 3. Associations Between Maternal and Child's Exposure to Narrow and Broad Spectrum Antibiotics and Obesity and BMI *z*-Score at Age 4 Years

eTable 4. Classification of Antibiotics According to Spectrum of Activity

This supplementary material has been provided by the authors to give readers additional information about their work.

© 2020 Leong KSW et al. *JAMA Network Open.*

eFigure 1. Obesity Prevalence at Age 4 Years According to Number of Antibiotics Dispensed to Mother in the Pre-conception Period (3 Months Before Pregnancy)

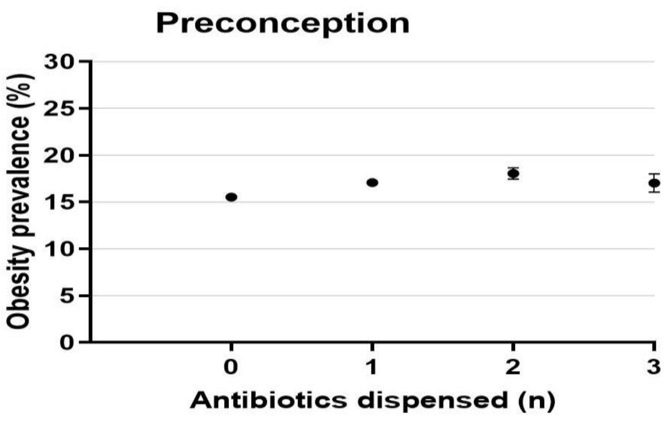

Data are unadjusted percentages with error bars representing the respective standard errors.

© 2020 Leong KSW et al. *JAMA Network Open.*

eFigure 2. Obesity Prevalence at Age 4 Years According to Number of Broad or Narrow Spectrum Antibiotics Dispensed

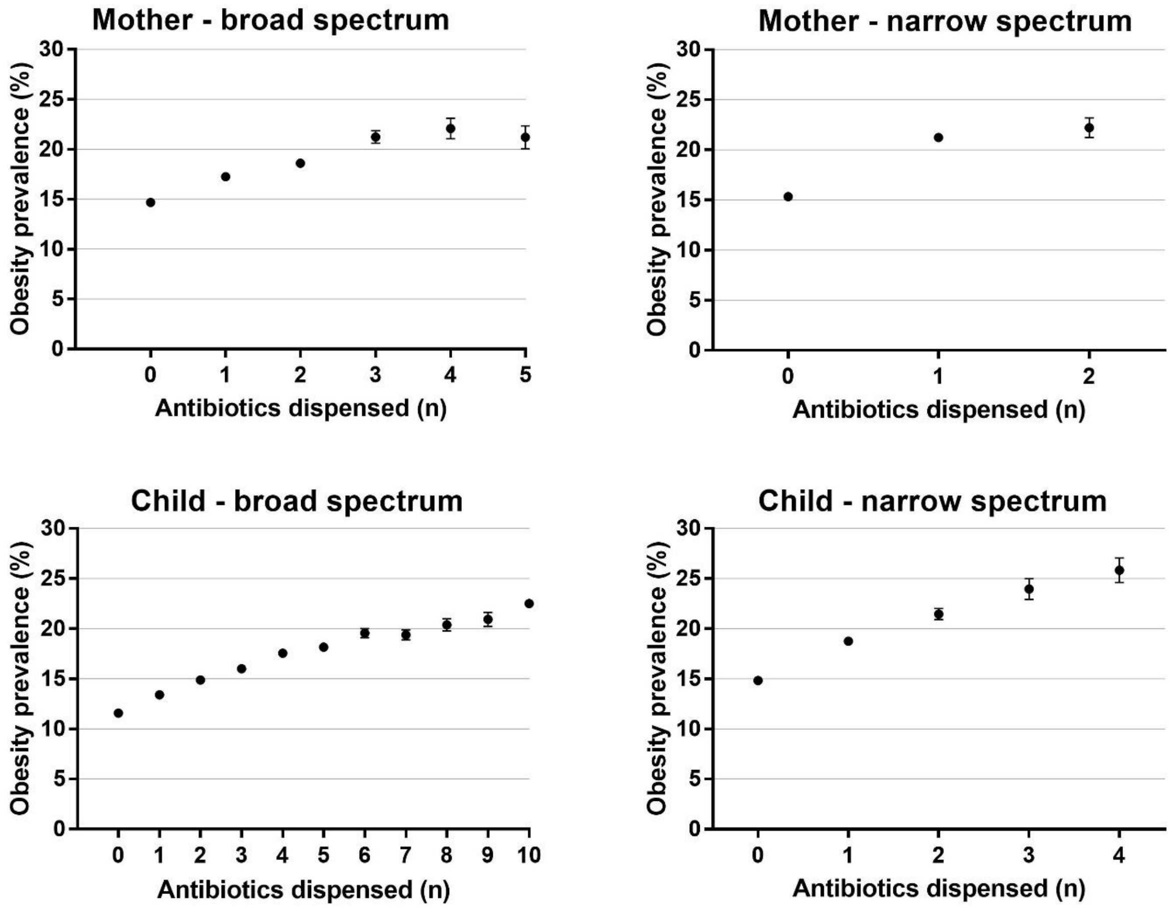

Data are unadjusted percentages with error bars representing the respective standard errors.

© 2020 Leong KSW et al. *JAMA Network Open.*

eTable 1. Demographic Characteristics of the Siblings (n=35 994) and Twins (n=4362) Cohort

		SIBLINGS	TWINS
**MOTHER**			
**Age at childbirth (years)**	<20	2,238 (6.2%)	174 (4.0%)
	20-24	7,497 (20.8%)	597 (13.7%)
	25-29	9,441 (26.2%)	912 (20.9%)
	30-34	10,326 (28.7%)	1,326 (30.4%)
	35-39	5,628 (15.6%)	1,104 (25.3%)
	≥40	861 (2.4%)	249 (5.7%)
**Parity**	First birth	13,404 (37.2%)	1,266 (29.0%)
	Later birth	22,590 (62.8%)	3,090 (70.8%)
**Socioeconomic status**	NZDep quintile 1 (least deprived)	6,501 (18.1%)	936 (21.5%)
	NZDep quintile 2	6,057 (16.8%)	846 (19.4%)
	NZDep quintile 3	6,246 (17.4%)	849 (19.5%)
	NZDep quintile 4	6,447 (17.9%)	798 (18.3%)
	NZDep quintile 5 (most deprived)	10,695 (29.7%)	924 (21.2%)
**Gestational diabetes**		1,068 (3.0%)	192 (4.4%)
**Hyperemesis gravidarum**		609 (1.7%)	195 (4.5%)
**3+ days in hospital during pregnancy**		1,395 (3.9%)	513 (11.8%)
**CHILD**			
**Year of birth**	2008	7,725 (21.5%)	738 (16.9%)
	2009	10,221 (28.4%)	1,398 (32.0%)
	2010	10,356 (28.8%)	1,491 (34.2%)
	2011	7,692 (21.4%)	729 (16.7%)
**Sex**	Male	17,439 (48.4%)	2,205 (49.4%)
	Female	18,555 (51.6%)	2,154 (50.6%)
**Ethnicity**	New Zealand European	24,492 (68.0%)	3,249 (74.5%)
	Māori	11,301 (31.4%)	1,170 (26.8%)
	Pacific	6,369 (17.7%)	567 (13.0%)
	Asian	2,844 (7.9%)	318 (7.3%)
	♦MELAA	420 (1.2%)	60 (1.4%)
	Other	543 (1.5%)	87 (2.0%)
**Birth weight**	Low birth weight (<2500g)	3,357 (9.3%)	2,160 (49.5%)
	Normal-high birth weight (≥2500g)	32,565 (90.5%)	2,193 (50.3%)
**Gestational age**	Very preterm (<32 weeks)	606 (1.7%)	363 (8.3%)
	Preterm (≥32 and <37 weeks)	3,561 (9.9%)	2,010 (46.1%)
	Term (≥37 and <42 weeks)	31,608 (87.8%)	1,980 (45.4%)
	Post-term (≥42 weeks)	216 (0.6%)	<6 (<0.1%)
**Delivery mode**	Unassisted vaginal	22,185 (61.6%)	909 (20.8%)
	Assisted vaginal	2,700 (7.5%)	648 (14.9%)
	Caesarean section	8,562 (23.8%)	2,673 (61.3%)
	Unknown	2,547 (7.1%)	129 (3.0%)
**Birth**	Singletons	31,587 (87.8%)	nil
	Twins or triplets	[1]4,407 (12.2%)	4,362 (100%)
**≥3 days in hospital in first 2 years of life**		4,941 (13.7%)	1,179 (27.0%)
**Obesity**		5,937 (16.5%)	510 (11.7%)

♦MELAA, Middle Eastern, Latin American or African

[1]This includes twins with siblings for whom no records exist for the co-twin.

© 2020 Leong KSW et al. *JAMA Network Open.*

eTable 2. Courses of Broad and Narrow Spectrum Antibiotics Dispensed to Mothers During Pregnancy, and to Children During the First 24 Months of Life

Mother, any time in pregnancy				Child, 0-24 months			
	⬩Broad spectrum		⬩Narrow spectrum		⬩Broad spectrum		⬩Narrow spectrum
**0**	102,825 (67.9%)	**0**	139,845 (92.4%)	**0**	30,228 (20.0%)	**0**	122,313 (80.8%)
**1**	30,750 (20.3%)	**1**	9,759 (6.4%)	**1**	26,562 (17.5%)	**1**	20,679 (13.7%)
**2**	10,878 (7.2%)	**2+**	1,755 (1.2%)	**2**	21,849 (14.4%)	**2**	5,439 (3.6%)
**3**	4,053 (2.7%)			**3**	17,091 (11.3%)	**3**	1,677 (1.1%)
**4**	1,617 (1.1%)			**4**	13,299 (8.8%)	**4+**	1,254 (0.8%)
**5+**	1,245 (0.8%)			**5**	10,065 (6.6%)		
				**6**	7,683 (5.1%)		
				**7**	5,820 (3.8%)		
				**8**	4,413 (2.9%)		
				**9**	3,411 (2.3%)		
				**10 +**	10,941 (7.2%)		

⬩Data are n (%).

© 2020 Leong KSW et al. *JAMA Network Open.*

eTable 3. Associations Between Maternal and Child's Exposure to Narrow and Broad Spectrum Antibiotics and Obesity and BMI *z*-Score at Age 4 Years

	[1]TYPE OF ANTIBIOTICS	⬩OBESITY aOR (95% CI)	▪BMI *Z*-SCORE β (95% CI)
**MOTHER**	Narrow spectrum	1.09 (1.06, 1.13)	0.053 (0.038, 0.067)
	Broad spectrum	1.04 (1.03, 1.06)	0.020 (0.015, 0.026)
**CHILD**	Narrow spectrum	1.05 (1.03, 1.07)	0.032 (0.026, 0.039)
	Broad spectrum	1.03 (1.02, 1.03)	0.013 (0.012, 0.015)

[1] Both types of antibiotics may have been prescribed; effects are not controlled for the presence of the other type of antibiotic.
aOR, adjusted odds ratios; BMI, body mass index.

⬩Data for obesity are adjusted odds ratios and 95% confidence intervals (CI)
▪Data for ▪BMI *z*-score are adjusted β coefficients and 95% confidence intervals
Reported estimates represent the effect of one additional course of antibiotics

Models adjusted for birth year and month, child sex and ethnicity, maternal age, parity, birth weight, gestational age, delivery mode, multiple birth status, maternal diabetes, and hyperemesis gravidarum, prolonged (≥3 days) stay in hospital during pregnancy and during the first 24 months of the child's life, neighbourhood deprivation, as well as number of days overseas during pregnancy (mother analyses only), and number of days overseas during the first 24 months of life (child analyses only).

© 2020 Leong KSW et al. *JAMA Network Open.*

eTable 4. Classification of Antibiotics According to Spectrum of Activity

Antibiotic category	⬩Proportions of antibiotics dispensed		Narrow	Broad
	mother	child		
Penicillins	31.2	78.1	Flucloxacilin<br>Penicillin V	Amoxycillin<br>Augmentin
Cephalosporins	3.7	13.4		Cefaclor<br>Cefalexin<br>Cefuroxime<br>Cephradine
Macrolides	7.4	15.3		Azithromycin<br>Clarithromycin<br>Erythromycin<br>Roxithromycin
Sulphonamides	3.1	20.0		Co-trimoxazole<br>Sulphadiazine
Others	12.4	nil	Fusidic acid<br>Vancomycin	Chloramphenicol<br>Ciprofloxacin

⬩ Data are percentages of at least one antibiotic prescription dispensed

© 2020 Leong KSW et al. *JAMA Network Open.*
